# An analysis of lawsuits relating to emergency physician performed point-of-care ultrasound

**DOI:** 10.1186/2036-7902-6-S1-A6

**Published:** 2014-01-31

**Authors:** KM O’Brien, LA Stolz, AM Gross, S Adhikari, M Blavais

**Affiliations:** 1 Massachusetts General Hospital, Boston, Massachusetts, USA; 2 University of Arizona, Department of Emergency Medicine, Tucson, AZ, USA; 3 Northside Hospital-Forsyth, Cumming, GA, USA

## Background

With any new technology, the opportunity arises for lawsuits related to the usage or failure to adopt the new technology. A previous study has analysed available records (1987 to 2007) for lawsuits related to point-of-care (POC) ultrasound (US).^1^ The use of POC US is rising, no studies have investigated the lawsuits related to POC US in the last 5 years.

## Objective

The aim of this study is to quantify and characterize lawsuits related to emergency physicians performing POC US.

## Patients and methods

The WESTLAW database is a repository of case law, state and federal statues, public records and other information sources used by legal professionals. The database was retrospectively reviewed for cases involving emergency physicians and US exams which fall under the ACEP core emergency US applications from January 2008 to August 2012. Cases were reviewed by emergency physicians with advanced US training. Cases were included if an emergency physician was named, the patient encounter was in the ED, the interpretation or failure to perform US was a central issue and the application was within the ACEP core applications.

## Results

120 cases returned from the database search and 8 cases met inclusion criteria. Most of these cases involved death or severe disability. Failure to perform within a timely manner or failure to perform studies was the most common allegation. The most common exam type in these cases was lower extremity venous US for detection of deep vein thrombosis.

## Conclusion

Between 1997 and 2013, there have been eight lawsuits documented in the Westlaw database which relate to POC US performed in the emergency department. There have been no documented cases of misinterpretation of POC US by an EP. Several cases relate to not performing a study or not obtaining a study in a timely manner.
